# Incorporating selected non-communicable diseases into facility-based surveillance systems from a resource-limited setting in Africa

**DOI:** 10.1186/s12889-019-6473-2

**Published:** 2019-02-04

**Authors:** A. O. Mocumbi, D. C. Langa, S. Chicumbe, A. E. Schumacher, W. K. Al-Delaimy

**Affiliations:** 1 Instituto Nacional de Saúde, 1008 Av. Eduardo Mondlane, Maputo, Moçambique; 2 Universidade Eduardo Mondlane, Maputo, Moçambique; 3 Hospital Geral de Mavalane, Maputo, Moçambique; 4 University of Washington, Seattle, USA; 5 University of California San Diego, San Diego, USA

**Keywords:** Non-communicable diseases, Health information system, Disease surveillance

## Abstract

**Background:**

As Mozambique faces a double burden of diseases, with a rise of Non Communicable Diseases (NCD) superimposed to uncontrolled communicable diseases (CD), routine disease surveillance system does not include NCD. The objectives of our study were to i) upgrade of the current surveillance system by adapting the data collection tools to NCD; ii) describe the occurrence and profile of selected NCD using these data collection tools.

**Methods:**

Workshops were implemented in a first referral urban hospital of Mozambique to train clinical staff, administrative workers and nurses on NCD surveillance, as well as select conditions to be prioritized. Based on the WHO Global Action Plan and Brazaville Declaration for NCD prevention and control, we selected arterial hypertension, diabetes, stroke, chronic respiratory diseases, mental illness and cancers. Data collection tools used for CD were changed to include age, gender, outcome and visit type. Between February/2014 and January/2015 we collected data at an urban hospital in Mozambique’s capital.

**Results:**

Over 12 months 92,018 new patients were assisted in this hospital. Data was missing or diagnosis was unreadable in 2637 (2.9%) thus only 89,381 were used for analysis; of these 6423 (median age 27 years; 58.4% female) had at least one selected NCD as their primary diagnosis: arterial hypertension (2397;37.31%), mental illness (1497;23.30%), asthma (1495;23.28%), diabetes (628;9.78%), stroke (299;4.66%), chronic obstructive pulmonary disease 61 (0.95%) and cancers 46 (0.72%). Emergency transfers were needed for 76 patients (1.2%), mainly due to hypertensive emergencies (31; 40.8%) and stroke (18;23.7%). Twenty-four patients died at entry points (0.3%); 10 of them had hypertensive emergencies.

**Conclusion:**

Changes in existing surveillance tools for communicable diseases provided important data on the burden and outcomes of the selected NCD helping to identify priority areas for training and health care improvement. This information can be used to design the local NCD clinics and to strengthen the health information system in resource-limited settings in a progressive and sustainable way.

**Electronic supplementary material:**

The online version of this article (10.1186/s12889-019-6473-2) contains supplementary material, which is available to authorized users.

## Background

A combination of broad and localized environmental factors, infectious disease and lifestyle behaviors determine the occurrence of deadly and disabling forms of communicable and non-communicable disease (NCD) in Africa. Mozambique, a low-income country in southern Africa, faces this double burden of disease characterized by uncontrolled endemic infections such as malaria, tuberculosis, human immunodeficiency virus/acquired immunodeficiency syndrome (HIV/AIDS) and neglected parasitic diseases, as well as high prevalence of risk factors for NCD [1–3]. Despite the top four causes of mortality being infectious diseases (Malaria, HIV/AIDS, Diarrheal diseases, Lower Respiratory Infections) and representing 57% of total deaths [4], the analysis of 8114 deaths classified in the autopsy register in country’s capital revealed that 22.6% were due to NCD [5]. The prevalence of hypertension nationwide in patients aged 25–64 years increased from 33.1 to 38.9% (p 0.048) between 2005 and 2015 [6]. Crude and adjusted (world reference population) annual incidence rates of stroke in Mozambique’s capital in 2009 were 148.7 per 100,000 and 260.1 per 100,000 aged ≥25 years, respectively [2]. Of 651 patients with new stroke events (mean age 59.1 ± 13.2 years and 53% men) 561 patients (86.2%) had prior hypertension; 28-day case-fatality was 49.6% (72.3% for hemorrhagic stroke), and 64.4% of 370 survivors at 28 days had moderate-to-severe disability [2].

The health information system (HIS) in Mozambique is largely concentrated on communicable diseases (CD). Systematic NCD notification does not exist, partially due to shortage of specialized clinical staff to ensure accurate diagnosis at peripheral health facilities [7], and NCD information available is restricted to aggregated hospital mortality [8].

Mozambique’s progress towards implementing the NCD global action plan 2013–2020 is off track. Despite the establishment of the NCD National Program within the Ministry of Health in the 2012 [9] NCD clinics are not functional. Lack of data on the burden and profile of NCD is a barrier to informed policy making, namely to design NCD clinics and implement the needed shifts in health professional training and resource allocation. The objectives of our study were to i) upgrade of the current surveillance system by adapting the data collection tools to NCD; ii) describe the occurrence and profile of selected NCD using these data collection tools.

## Methods

### Setting

*Mavalane General Hospital (MGH)* is a 239-bed hospital in the capital of Mozambique, serving nearly 800,000 people. It represents the prototype of secondary level hospitals in the country, which in Mozambique’s health system are the first referral hospitals capturing transfers from surrounding health centres, and providing beds for four specialties (Internal Medicine, Surgery/Traumatology, Pediatrics and Gynecology-Obstetrics). The National Public Health Institute (*Instituto Nacional de Saúde, INS*) holds the mandate to implement research that informs local health policy, and has adopted MGH for surveillance of endemic diseases (malaria, tuberculosis, HIVAIDS, parasitic diseases, diarrheal diseases) using tools approved by the Ministry of Health (MoH) to feed the Information System on Diseases of Compulsory Declaration. Daily consultations performed by doctors and clinical officers are registered in books from where nurses and administrative workers routinely extract data on CD targeted by national programs, to produce weekly reports that are centralized at the MoH. Because patient electronic management system is not available, identification cards are provided to patients to allow recovery of medical files for follow up.

### Data collection tools

Registration books used for consultations at entry points included patient’s name, age group (0–4 years; 5–14 years; > 15 years), diagnosis, treatment provided, type of visit (first or follow up) and observations (Additional file 1). For our study we proposed to replace age group by the exact patient’s age, to add and extra column with gender, and to use the observation’s column to register the immediate outcome (discharge, transfer or death).

### Data collectors training

**Fig. 1:**
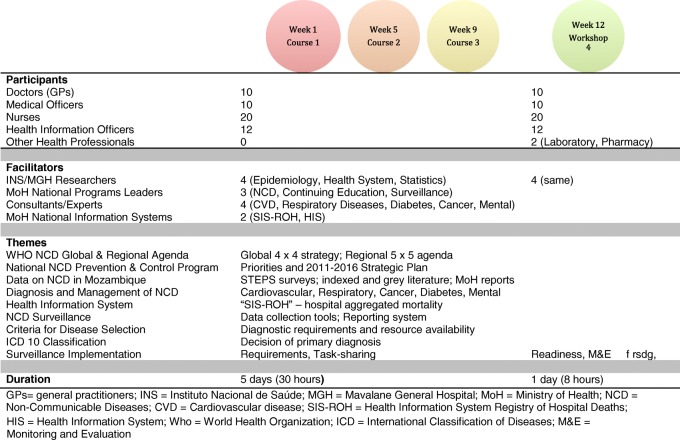
Training scheme, cadres involved and themes discussed and at MGH

All clinicians working in triage, out-patients clinics and emergency department, as well as administrative workers involved in surveillance attended a 5-days (30 h) course on NCD diagnosis, management and surveillance. Twenty doctors/clinical officers, 20 nurses and 12 administrative attended the course in groups of 16–18 health workers, to avoid stopping all clinical activities. INS researchers (AM, DC, SC) designed the training program and delivered with the support of leadership from the NCD National Program, senior researchers at INS and consultant specialists from relevant areas (Fig. 1). The three workshops were delivered over the 3 months prior to the study. Formal training and group discussions were used to discuss the topics and the hospital readiness to report on diseases targeted by the WHO Global Action Plan for the prevention and control of NCD 2013–2020 [10], prioritized in the Mozambique’s NCD Control Program [9] and adopted by the African Governments [11], but also to identify other conditions of interest to the group. Considering research data available, human resources available, diagnostic capacity and health worker’s perception of clinical relevance, we then selected the conditions that would be targeted for surveillance, namely: arterial hypertension, asthma, cancer, chronic obstructive pulmonary disease, diabetes, mental illnesses (depression, psychosis, epilepsy and alcohol abuse) and stroke [1–3, 12–19] (Table 1). One week before the starting of data collection a one-day workshop was delivered separately to clinicians – to review the updated registration books and the use of the international classification of diseases (ICD-10) [https://en.wikipedia.org/wiki/ICD-10] – and to administrative workers and nurses – to train data extraction from registration books, building of daily reports, and use an Epi Info V.7 database for data entry. At this point a monitoring and evaluation plan was also agreed.

**Table 1:** Priority-setting criteria for NCD surveillance at first referral hospital. We considered all conditions included in the Mozambique’s NCD Control Plan and added COPD and Mental Illnesses

Condition	Data Available	Rational for Choice	MGH Readiness
Arterial Hypertension	33% prevalence in adults nationwide 2005 [1] 39% prevalence in adults nationwide 2015 [3] Low rate of control	High burden; high cost for the individual and the health system; high morbidity and mortality	Easily recognizable; Initial target of national action plan
Stroke	1.7 strokes/day with high mortality rate [2]	High morbidity and mortality; high cost for the individual and the health system; Poor management	Easily recognizable
Diabetes	2.9% prevalence in adults nationwide 2005 [3] 7.8% prevalence in adults nationwide 2015 Low rate of control	High burden; high morbidity and mortality; high cost to the individual and the health system; Poor management	Tools available for diagnosis; Initial target of national action plan
Cancers	Cervical first cause of cancer in women [12, 13] Prostate Breast	High burden on national registries Preventable infection with vaccine Screening available; Poor management	VIA test in place Easily recognizable PSA Mammography
Asthma	13.3% prevalence in 67y and 13-14y [14] 2nd reason for in-patient treatment in Maputo	High burden on individuals and health system; high morbidity; Poor management	Easily recognizable; Target of national action plan
Chronic Obstructive Pulmonary Disease	No data available in the literature [15]	High use of biomass fuels; Need to assess role of occupational health; High prevalence of pulmonary tuberculosis	Tools available for diagnosis
Mental Illnesses	Alcohol abuse [16] Epilepsy [17, 18] Depression/Suicide [19] Psychosis	High burden; High proportion of hospital admissions; High cost on individual and health system; Poor management	Target of national action plan; doctors & mental health clinical officers deployed;

*PSA* Prostatic Specific Antigen, *VIA* visual inspection with acetic acid

### Data collection procedures

We prospectively collected data on all patients attending MGH between 1st February 2014 and 31st January 2015. INS researchers based at MGH were distributed by the different sectors of the hospital to i) provided support to those involved in data capture; ii) reviewed patient’s files and checked data sources to ensure data quality; iii) helped in defining the primary diagnosis when multiple conditions were listed. For logistical reasons we only entered in the database full data from patients with primary diagnosis of a selected NCD. To avoid duplication of entries the hospital identification cards of patients with selected NCD were labeled with a unique study identification number.

### Monitoring and evaluation

AM, DC and SC compiled data from all sectors weekly (all patients seen in hospital), and provided the health workers and hospital leadership with feedback on progression of the study and discussed areas for improvement on a monthly basis.

### Statistical analysis

We performed descriptive analysis of patients with selected NCD. Age is presented as median and categorized in three groups: children (0–17), adults (18–64) and elderly (> 65). For gender, diagnosis and outcomes data is presented as percentages and proportions.

### Ethical issues

The national bioethics committee (IRB 000002657) approved the study.

## Results

**Fig. 2:**
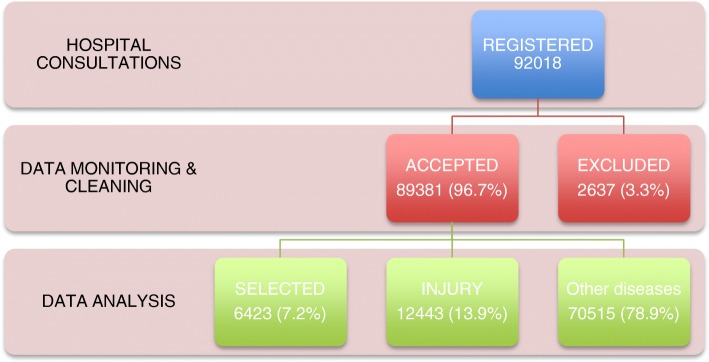
Summary of patients registered at MGH over 12 months with breakdown of traumatic, non-traumatic patients diagnosed with selected conditions and other patients see seen at entry points. “INJURY” includes physical injury and poisoning (except if related to suicidal attempt, in which case it was considered mental illness); “OTHERS” include CD and other non-communicable diseases that were not selected for surveillance

Over 12 months 92,018 patients were registered at entry points at MGH; of these the diagnosis was missing, unreadable or could not be confirmed in 2637 (2,9%). The selected NCD were found in 6423 (7.2%) of the 89,381 patients considered for analysis. Overall, 9290 (10.4%) patients were admitted to wards, 3398 (3.8%) died and 1047 (1.2%) were transferred for specialized care (Fig. 2).

### Burden and profile of the selected NCD

The 6423 patients with NCD had median age 27 years and 58.4% were females. The diseases were distributed as follows: arterial hypertension (2397;37.31%), mental illness (1497;23.30%), asthma (1495;23.28%), diabetes (628;9.78%), stroke (299;4.66%), chronic obstructive pulmonary disease 61 (0.95%) and cancers 46 (0.72%).

**Fig. 3:**
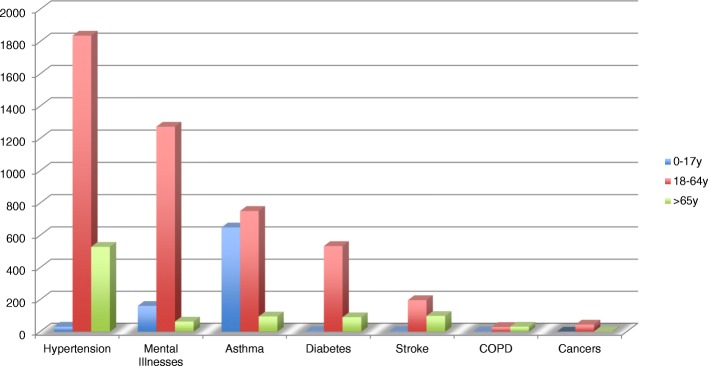
Distribuition of selected NCD by age groups

Most patients were adults aged 18–64 (4665; 72.6%). The elderly (> 65 years) were proportionally more represented for chronic obstructive pulmonary disease (50.8%) and stroke (32.8%), while children and adolescents represented 43.4% of patients with asthma. Cancer was more frequent in adults 18–64 years (44 out of the 46 cases). The age distribution of patients for each selected condition is presented in Fig. 3.

Female predominance was marked for arterial hypertension (68,3% females vs 31.7% males) and depression (65.5% females vs 44.5 males), while there was male predominance for alcohol abuse (66.1% males vs 33.9% females). Table 2 shows gender distribution for each selected condition.

**Table 2:** Frequency of selected NCD by gender, with mental illness disaggregated by the diagnosis considered in the study

Condition	Female (%)	Male (%)	Total
Hypertension	1638 (68.3)	759 (31.7)	2397 (37.31)
Mental Illnesses	754 (50.4)	743 (49.6)	1497 (23.30)
*Psychosis*	*189 (52.6)*	*170 (47.4)*	*359*
*Epilepsy*	*244 (48.6)*	*258 (51.4)*	*502*
*Depression*	*218 (65.5)*	*115 (34.5)*	*333*
*Alcohol abuse*	*103 (33.9)*	*200 (66.1)*	*303*
Asthma	756 (50.6)	739 (49.4)	1495 (23.28)
Diabetes	394 (62.7)	234 (37.3)	628 (9.78)
Stroke	165 (55.2)	134 (44.8)	299 (4.66)
COPD	28 (45.9)	33 (54.1)	61 (0.95)
Cancers	18 (39.1)	28 (60.9)	46 (0.72)
Total	3753 (58.4%)	2670 (41.6%)	6423 (100%)

*COPD* Chronic Obstructive Pulmonary Disease

### Outcomes

Emergency transfers were needed for 76 patients (1.2%) patients with selected NCD. The most common causes were hypertensive emergencies (31; 40.8%) and stroke (18; 23.7%). Of the 24 patients that died at entry points (0.3%) 16 were aged 18 to 64 years and 10 had hypertensive emergencies.

## Discussion

This study provided a model to incorporate NCD surveillance into an existing HIS directed to CD. Our results reveal for the first time facility-based data on occurrence and profile of NCD in Mozambique, which is essential to inform policy and promote health system responses to address the growing threat represented by these conditions in this low-income country with shortage of human resources. Hypertension, chronic respiratory diseases and mental illnesses constituted 84% of selected NCD in this predominantly young population. Finally, the gender- and age-specific patterns provide valuable data for using patient-centered approaches in designing local NCD clinics.

WHO supported Stepwise Approach to Surveillance (STEPS) surveys [1, 3, 16] have provided initial data on prevalence of risk factors for chronic diseases in the country. Our study is an attempt to complement data from these community-based surveys, by providing information on service demand and burden at first referral level of the health system. Our approach included training of health professionals, selection of diseases to target and tailoring of existing data collection tools because, like in may under-resourced settings in Africa, NCD surveillance has never been done in our hospital. Additionally, due to task-shifting of specialist’s skills to general practitioners and clinical officers in our setting, we though that an introductory course on diagnosis and surveillance would equip all involved with the necessary knowledge to ensure quality of diagnosis. Moreover, by addressing the institutional surveillance capacities to support chronic care and high demand – two major barriers to NCD surveillance in under-resourced settings – with the health workers involved in care provision and surveillance we expect to create a sense of ownership and community engagement.

Loss of information in 2.9% patients occurred despite continuous support by INS researchers, but was higher during the first 3 months of the study. Overall we were able to capture full data from over 97% patients. In South Africa factors that influenced correct disease notification by health care providers were their perceptions of workload (OR 0.84, 95% CI 0.70–0.99, *p* = 0.043) and usefulness of the information collected (OR 0.84, 95% CI 0.71–0.99, *p* = 0.040) [20]. Considering the high workload of health care providers at MGH and the diversity in their background, we implemented continuous supervision and frequent feedback meetings. However, no association was found between correct notification and experience or training on disease surveillance, understanding of the purpose of the surveillance system, or even perception of feedback given to health care providers [20], and thus qualitative research would be advisable to understand the reasons behind our findings.

Despite the establishment of the NCD National Program within the Ministry of Health in 2012 [9], Mozambique’s progress towards implementing the NCD global action plan 2013–2020 is off track. NCD clinics are not functional at health facilities and cause–specific NCD morbidity and mortality has not been incorporated in the national health reporting system, partially due to the need for multiple approaches to collect comprehensive information on NCD surveillance. In Africa despite 91% of the 54 countries having established NCD units within the Ministries of Health in 2011, only 32% of these were functional in 2014 [http://www.who.int/nmh/publications/en/]. South Africa was the only country to have set time-bound national NCD indicators, and to address NCD mortality and the key risk factors in the 2015 report [21].

Despite the Global Burden of Disease indicating that a large proportion of NCD disability-adjusted life year in Mozambique is not attributable to known risk factors [22], we suggest that the existing framework of four major NCD (cardiovascular diseases, cancer, diabetes and chronic respiratory diseases) and their four risk factors (tobacco use, unhealthy diet, physical inactivity and harmful use of alcohol) [10] be used to promote change. We therefore selected conditions that are prioritized by the National Strategic Plan for NCD prevention and control [9], and included mental health has proposed by Mayosi and Mensah [23]. We strongly believe that chronic complications of infections (eg. tuberculosis, HIV/AIDS, schistosomiasis, cystecercosis) and endemic neglected diseases (eg. rheumatic, heart disease, sickle cell disease, cardiomyopathies, epilepsy, Burkitt Lymphoma, etc) should be progressively included in NCD surveillance systems according to the country’s disease profile. This is important to provide comprehensive care to infectious diseases and to promote equity in health care provision.

We acknowledge that facility-based surveillance only captures patients with access to, the health system and may vary according to the geographic setting and diagnostic capacity at the facilities. HIV surveillance has been shifting over the past two decades from reliance on sentinel surveys and AIDS case reporting to include household surveys, community-based surveys, and HIV case reporting [24]. The use of mixed (facility- and community-based) methods for NCD surveillance in Africa may also be useful to confirm unique patterns suggested by single center or geographically restricted studies on cardiovascular [25, 26], respiratory [27] and psychiatric [17] diseases.

Our results influenced the design of NCD services in MGH’s catchment area and the creation of management algorithms to be used by non-specialists as part of decentralization of NCD care. Additionally, improvements to data collection tools to allow patient follow up (patients residence and contacts) are now being tested in other regions of Mozambique.

## Conclusions

Changes in existing surveillance tools for communicable diseases provided important data on the burden and profile of the selected, non-communicable diseases helping to identify priority areas for training and health care improvement. This information can be used to design clinics for chronic diseases, progressively strengthen the health information system, and support evidence-based resource allocation to address the double burden of diseases in resource-limited settings. Research is warranted to explore the sustainability and cost of this strategy.

## Additional file

Additional file 1: Registration Books. Image of the registration books used for the study. Marked in red is the “age” column, which was changed from “age group”. In green are the new columns (gender and immediate outcome) added to the original data collection form. (DOCX 55 kb)

## Abbreviations

CD: Communicable diseases
HIS: Health Information System
INS: Instituto Nacional de Saúde
MGH: Mavalane General Hospital
MoH: Ministry of Health
NCD: Non-communicable diseases
STEPS: Stepwise approach to surveillance
WHO: World health organization
