# Supplementary material for: Incorporating selected non-communicable diseases into facility-based surveillance systems from a resource-limited setting in Africa
Source: BMC Public Health. 2019 Feb 4;19:147. doi: 10.1186/s12889-019-6473-2 (PMC6360799; doi:10.1186/s12889-019-6473-2)
Supplement: Supplementary file 1 — Registration Books. Image of the registration books used for the study. Marked in red is the “age” column, which was changed from “age group”. In green are the new columns (gender and immediate outcome) added to the original data collection form. (DOCX 55 kb) [file 12889_2019_6473_MOESM1_ESM.docx]

**SUPPLEMENTARY FIGURE**

| Monthly Nr | Name | AGE | GENDER | Diagnosis | Management | Visit Type | Observations  OUTCOME |
| --- | --- | --- | --- | --- | --- | --- | --- |
|  |  |  |  |  |  |  |  |
|  |  |  |  |  |  |  |  |
|  |  |  |  |  |  |  |  |
